# Variables Associated With Favorable Obstetrical Outcomes in Early Pregnancy Bleeding in the Emergency Department

**DOI:** 10.7759/cureus.70986

**Published:** 2024-10-07

**Authors:** Morgan M Burgoyne, Robin Clouston, Ankona Banerjee, Kavish Chandra, Jacqueline Fraser, David Lewis, Paul Atkinson

**Affiliations:** 1 Obstetrics and Gynecology, Dalhousie University, Halifax, CAN; 2 Family Medicine/Emergency Medicine, Dalhousie Family Medicine, Saint John, CAN; 3 Pediatrics/Epidemiology, Baylor College of Medicine, Houston, USA; 4 Emergency Medicine, Dalhousie Medicine New Brunswick, Saint John, CAN; 5 Emergency Medicine, Horizon Health Network, Saint John, CAN; 6 Emergency Medicine, Saint John Regional Hospital, Saint John, CAN; 7 Emergency Medicine, Dalhousie University, Halifax, CAN

**Keywords:** emergency medicine, first trimester bleeding, live birth rate, obstetric outcome, pocus (point of care ultrasound)

## Abstract

Background

Vaginal bleeding in early pregnancy is a frequent emergency department presentation and is associated with various obstetrical outcomes. Despite this, there has been limited research on the variables that predict less-than-preferred obstetrical outcomes in these cases. This study aims to identify predictors of preferred obstetrical outcomes for women presenting to the emergency department with early pregnancy bleeding.

Methods

We conducted a retrospective review of health records from an emergency department at a Canadian tertiary care center. Pregnant females presenting with vaginal bleeding before 20 weeks gestation were included. Variables analyzed included maternal age, gravidity, parity, hemoglobin levels, Rh status, cramping, socioeconomic status, and ultrasound findings. The primary outcome was a preferred outcome, defined as a full-term live birth (≥37 weeks). Less-preferred outcomes included miscarriage, preterm birth, and stillbirth. Point-of-care ultrasound and radiology ultrasound findings were also evaluated.

Results

A total of 422 patients were screened, and 180 were included in the analysis. Overall, 75 (41.7%) patients had a preferred outcome, while 105 (58.3%) had a less-preferred outcome. The strongest predictor of a preferred outcome was the presence of a live intrauterine pregnancy with fetal heartbeat on ultrasound, with a preferred outcome rate of 74.5% (56/76) (95% CI 59.8-88.7; p < 0.01) on point-of-care ultrasound (POCUS), and 100% (65/65); p = 0.04 for radiology-performed ultrasound. In contrast, 80.8% (21/26) of patients with findings other than a live intrauterine pregnancy on POCUS, and 100% (88/88) on radiology-performed ultrasound had a less-preferred outcome. Cramping with bleeding was associated with a higher rate of less-than-preferred outcomes (62.1%, 77/124; 95% CI 54.2-71.1; p = 0.07). Socioeconomic status was not a significant predictor, with similar outcomes above and below the poverty line. Anemia was associated with a 100% non-live birth rate, although only 5 patients were anemic.

Conclusion

Identification of a live intrauterine pregnancy on ultrasound is a strong predictor of a preferred outcome in early pregnancy bleeding. POCUS has the advantage of being immediately available, whereas radiology-performed ultrasound may be more definitive as a predictor. Cramping and anemia may also be associated with less-preferred outcomes, though further research is needed to confirm these findings. These predictors may help guide clinical decision-making and improve counseling for patients presenting to the emergency department with early pregnancy bleeding.

## Introduction

Vaginal bleeding in early pregnancy is a common reason for presentation to the emergency department, with an estimated 20-40% of pregnant women experiencing bleeding in the first trimester [1]. While many cases result in positive outcomes, approximately 50% of pregnancies complicated by early bleeding end in miscarriage [2]. Early pregnancy bleeding is also associated with other adverse outcomes, such as preterm birth, preterm premature rupture of membranes, and fetal growth restriction [3].

The use of point-of-care ultrasound (POCUS) in the emergency department setting has become more prevalent, allowing clinicians to quickly assess the viability of a pregnancy, although the use of radiology-performed ultrasound remains embedded in many early pregnancy protocols. Previous studies have shown that the presence of a live intrauterine pregnancy on POCUS in the second half of pregnancy is a strong predictor of live birth [4]. However, there has been limited research on the predictive value of ultrasound findings and other clinical variables in the first half of pregnancy, particularly in women presenting with early pregnancy bleeding.

This study aims to identify predictors of preferred (defined as a full-term live birth (≥37 weeks) and less-preferred obstetrical outcomes (defined as miscarriage, preterm birth, or stillbirth) for women presenting to the emergency department with early pregnancy bleeding before 20 weeks gestation. We hypothesize that specific variables, including the presence of a live intrauterine pregnancy on ultrasound, maternal age, cramping, anemia, and socioeconomic status, may be associated with either preferred or less preferred outcomes.

## Materials and methods

Study design and population

This retrospective cohort study was conducted at a Canadian tertiary care center emergency department. The study population included pregnant women presenting with vaginal bleeding before 20 weeks gestation. Patients were excluded if they presented with trauma, had already completed a miscarriage, or had undergone a therapeutic abortion. The primary outcome was a preferred outcome, defined as a full-term live birth (≥37 weeks). Less-preferred outcomes included miscarriage, preterm birth, or stillbirth.

Data collection

Patient records were reviewed to extract demographic information, clinical characteristics, and outcomes. Predictor variables included maternal age, gravidity, parity, hemoglobin levels, Rh status, cramping, number of return emergency department visits, and socioeconomic status. The socioeconomic status was inferred from the patient’s postal code using public data on income levels. Hemoglobin levels were categorized as normal (≥110 g/L) or anemic (<110 g/L).

Ultrasound data

POCUS results and formal radiology ultrasound findings were recorded. The presence or absence of a live intrauterine pregnancy with a fetal heartbeat was documented. Patients with live intrauterine pregnancy on ultrasound were classified into the preferred outcome group if they delivered a live baby at ≥37 weeks.

Statistical analysis

Descriptive statistics were used to summarize the data, and chi-square tests were employed to compare categorical variables. Continuous variables were analyzed using t-tests. A p-value <0.05 was considered statistically significant. A 95% confidence interval was calculated for proportions.

## Results

Study population

Of the 422 patients screened, 180 met the inclusion criteria. Of these, 75 patients (41.7%) had a preferred outcome, defined as a full-term live birth. A total of 105 patients (58.3%) experienced a less-preferred outcome, including miscarriage (n=53, 29.4%), preterm birth (n=45, 25%), and stillbirth (n=7, 3.9%).

Predictors of obstetrical outcomes

Ultrasound

The strongest predictor of a preferred outcome was the presence of a live intrauterine pregnancy (IUP) with fetal heartbeat on ultrasound. Among the 76 patients with a live intrauterine pregnancy on POCUS, 56 (73.7%) had a preferred outcome, while 20 (26.3%) had a less preferred outcome (p < 0.01). In contrast, 80.8% (21/26) of patients with other findings on POCUS (no clear live IUP) had a less preferred outcome, with only 5 (19.2%) achieving a preferred outcome. Of the 153 patients who underwent a radiology-performed ultrasound scan, 100% (65/65)) of those where a live IUP was demonstrated on ultrasound proceeded to have a preferred obstetrical outcome. In addition, none (0%; 0/88) of the patients with radiology-performed ultrasound findings other than a definitive live IUP went on to have a preferred outcome (p =0.04).

Cramping and Bleeding

Among the 124 patients who reported cramping with bleeding, 47 (37.9%) had a preferred outcome, while 77 (62.1%) had a less-preferred outcome (p = 0.07). Cramping was thus associated with a trend toward poorer outcomes, though this did not reach statistical significance.

Socioeconomic Status

Socioeconomic status was not a significant predictor of outcomes. Among patients above the poverty line, 41% (41/100) had a preferred outcome, compared to 42.5% (34/80) of patients below the poverty line (p = 0.84).

Hemoglobin Levels

Anemia was associated with poorer outcomes, although the number of anemic patients was small (n=5). All 5 anemic patients (100%) experienced a less-preferred outcome.

Rh Status and Other Variables

Rh status was not a significant predictor, with similar outcomes between Rh-positive (41/142, 28.9%) and Rh-negative patients (20/38, 52.6%, p = 0.83). Gravidity, parity, and maternal age were also not associated with a significant difference in outcomes. All results are summarized in Table 1.

**Table 1:** Maternal and Clinical Characteristics, Ultrasound Findings, and Obstetrical Outcomes in Women Presenting with Early Pregnancy Bleeding (n = 180) “Preferred outcome” refers to a full-term live birth (≥37 weeks); “Less-preferred outcome” refers to miscarriage, preterm birth, or stillbirth. The p-values indicate the statistical significance of comparisons between preferred and less-than-preferred outcomes for each variable. The data are presented as numbers (%) to provide clear insights into the distribution of variables.

Variable	n (%)	Preferred Outcome (≥37 weeks)	Less-Preferred Outcome	p-value
Maternal Age (years)				
15-24	53 (29.4%)	23 (43.4%)	30 (56.6%)	0.75
25-29	45 (25%)	19 (42.2%)	26 (57.8%)	
30-34	54 (30%)	24 (44.4%)	30 (55.6%)	
35-44	28 (15.6%)	9 (32.1%)	19 (67.9%)	
Gravida				
One	50 (27.8%)	20 (40%)	30 (60%)	0.64
Two or more	130 (72.2%)	55 (42.3%)	75 (57.7%)	
Parity				
Zero	103 (57.2%)	41 (39.8%)	62 (60.2%)	0.48
One or more	77 (42.8%)	34 (44.2%)	43 (55.8%)	
Hemoglobin Levels				
Normal (≥110 g/L)	175 (97.2%)	75 (42.9%)	100 (57.1%)	0.08
Anemic (<110 g/L)	5 (2.8%)	0 (0%)	5 (100%)	
Cramping/Abdominal Pain				
Present	124 (68.9%)	47 (37.9%)	77 (62.1%)	0.07
Absent	56 (31.1%)	28 (50%)	28 (50%)	
Rhesus Status				
Rh positive	142 (79%)	55 (38.7%)	87 (61.3%)	0.83
Rh negative	38 (21%)	20 (52.6%)	18 (47.4%)	
Socioeconomic Status				
Above poverty line	100 (55.6%)	41 (41%)	59 (59%)	0.84
Below poverty line	80 (44.4%)	34 (42.5%)	46 (57.5%)	
Ultrasound Findings				
Point-of-care ultrasound performed	102 (56.7%)	76 (74.5%)	26 (25.5%)	<0.01
Live intrauterine pregnancy	76 (74.5%)	56 (73.7%)	20 (26.3%)	<0.01
Other	26 (25.5%)	5 (19.2%)	21 (80.8%)	
Radiology Ultrasound Findings				
Performed	153 (85.1%)	65 (42.5%)	88 (57.5%)	
Live intrauterine pregnancy	65 (42.5%)	65 (100%)	0 (0%)	0.04
Other	88 (57.5%)	0 (0%)	88 (100%)	
Pregnancy Outcome				
Full-term live birth (≥37 weeks)	75 (41.7%)	75 (100%)	-	-
Preterm birth	45 (25%)	-	45 (100%)	-
Miscarriage	53 (29.4%)	-	53 (100%)	-
Stillbirth	7 (3.9%)	-	7 (100%)	-

## Discussion

This study demonstrates that the presence of a live intrauterine pregnancy with fetal heartbeat on ultrasound is a strong predictor of preferred outcomes in women presenting to the emergency department with early pregnancy bleeding. Our findings align with previous research indicating that ultrasound findings in the second half of pregnancy are predictive of live birth [4], and extend these findings to the first half of pregnancy. Radiology-performed ultrasound was the strongest predictor of outcome, performing with strong accuracy in this sample; however, this service may not always be immediately available at the time of presentation. As such, it is worth noting that emergency physician-performed POCUS was also a strong predictor of obstetric outcome, and may be more readily accessible during the emergency department visit.

Cramping with bleeding was associated with a trend toward poorer outcomes, though this relationship did not reach statistical significance. These findings suggest that cramping may indicate an increased risk for less-preferred outcomes, and further research is warranted to clarify this association. Similarly, anemia was associated with 100% less preferred outcomes in our cohort, but the small sample size limits the generalizability of this finding. Previous studies have shown that anemia in pregnancy is associated with adverse outcomes, including preterm birth and low birth weight [5], and our results support this association.

Socioeconomic status was not found to be a significant predictor of obstetrical outcomes, suggesting that socioeconomic factors alone may not influence outcomes in this population. This contrasts with other studies that have demonstrated a relationship between lower socioeconomic status and adverse pregnancy outcomes [6]. This difference may be attributable to the universal healthcare system in which this study was conducted, which may reduce disparities in access to care.

Limitations

The addition of several other variables such as analysis of systemic comorbidities, the cause of the initial bleeding the patient presented with, identification of any history of gynecological comorbidities, and any previous obstetrical history or outcomes that could impact the current pregnancy, would have been helpful and may have impacted the accuracy of any of the analyzed predictors of outcome. Unfortunately, those variables were not available in our data set and as such could not be analyzed.

Clinical implications

It remains difficult to reliably predict preferred and less-than-preferred obstetric outcomes in patients presenting to the ED with early pregnancy bleeding. That said, by incorporating the use of both POCUS and radiology-performed ultrasound, as local resources permit [7], alongside other potentially useful variables as outlined above, a safe and compassionate approach can be taken to support the care of this potentially vulnerable group of patients [8]. It is important to establish pathways that can rapidly provide the information and counseling that is required.

## Conclusions

This retrospective study supports the finding of the presence of a live intrauterine pregnancy with fetal heartbeat on ultrasound as the most reliable predictor of preferred outcomes in early pregnancy bleeding presenting to the emergency department. Point-of-care ultrasound has the advantage of being immediately available, whereas radiology-performed ultrasound may be more definitive as a predictor. As such, they should be used in a complementary fashion. Anemia and cramping may also serve as important clinical indicators of less-preferred outcomes. These findings provide valuable insights for emergency department physicians managing early pregnancy bleeding and may help guide counseling and clinical decision-making.
